# Microstructure Evolution of Immiscible PP-PVA Blends Tuned by Polymer Ratio and Silica Nanoparticles

**DOI:** 10.3390/polym10091031

**Published:** 2018-09-17

**Authors:** Xiang Yan, Aurélie Cayla, Eric Devaux, Fabien Salaün

**Affiliations:** 1 Université Lille Nord de France, F-59000 Lille, France; aurelie.cayla@ensait.fr (A.C.); eric.devaux@ensait.fr (E.D.); fabien.salaun@ensait.fr (F.S.); 2 Ecole Nationale Supérieure des Arts et Industries Textiles (ENSAIT), GEMTEX, 2 allée Louise et Victor Champier, F-59100 Roubaix, France

**Keywords:** polypropylene, poly(vinyl alcohol), polymer blends, nano-silica, microstructure

## Abstract

Composites of polypropylene (PP) and water soluble poly(vinyl alcohol) (PVA) can become an environmentally friendly precursor in preparing porous material, and their biphasic morphology needs to be manipulated. In this work, PP-PVA extrudates were prepared with a twin-screw extruder, and different PP/PVA ratios were employed to manipulate the morphology of the blends. Afterwards, different silicas were imbedded within the blends to further regulate the biphasic microstructure. PVA continuity, as a vital parameter in obtaining porous material, was determined by selective extraction measurement, and PP-PVA biphasic morphology was characterized by scanning microscopy analyses (SEM). Rheological measurement was also performed to correlate the microstructure evolution of the blends. First, it was found that with the increment of PVA proportion, PVA continuity is raised gradually, and the microstructure of blends containing 40–50 wt % of PVA is approaching co-continuous. Second, the localization of silicas was predicted based on the wettability of silica and polymers, and it was also confirmed by TEM that different silicas showed selective distribution. It is inspiring that R972 nanoparticles were found mainly distributed at the interface, which gives a possibility in preparing a surface-modified porous material. The shape distribution and average size of PVA nodules were examined by analyzing the SEM images. It is indicated that silicas with different wettabilities play disparate roles in tuning the biphasic microstructures, leading to heterogeneous PVA continuity.

## 1. Introduction

Polymer blending has been an industrialized strategy to obtain new materials with excellent combined functions. With the development of polymer science, many kinds of water soluble phases can be selected, for example, poly(vinyl pyrrolidone) (PVP), poly(ethylene oxide) (PEO) [1] and poly(vinyl alcohol) (PVA). It offers an environmentally friendly method to fabricate porous material, and the sacrificial phase can be easily removed from water instead of organic solvents. Thus, the polymer pairs can also become an ideal precursor in preparing green porous material. Polymer blends are normally immiscible due to the low mixing entropy of polymers [2], and their morphology includes matrix/droplet, matrix/fiber, lamellar, and co-continuous structure [3]. Among the different microstructures, matrix/droplet [4,5] and *co*-continuous structure [6,7] are most noticeable, and sometimes the structure can be complicated and multifarious. Therefore, it is fascinating to control the microstructure to obtain the porous materials with a desired structure. Evolution of the porous microstructure mainly originates from the multiphase regulation of the polymer blends, and the most direct method is adjusting the polymer fractions [8].

Furthermore, isothermal treatment [9], incorporation of compatibilizers [10], etc. can also be adopted. Traditional compatibilizers are polymeric reagents, and have experienced a long-term development, derived into several species, including copolymers (block, graft, random) or reactive polymers [11]. With the development of nanotechnology, more commercialized nanoparticles are being utilized to tailor the microstructure. For instance, silica nanoparticles, as typical spherical particles, have been applied into polymer blending for many years. For its high specific surface area, even a very low fraction of silicas will alter the biphasic morphology significantly. Apart from regulating the structure, nanoparticles can endow the material with additional properties, for instance, elastic, barrier, thermal, and fire properties [12]. In the case that the nanoparticles are localized at the interface, after the etching of sacrificial phase, surface modified scaffold with nanoparticles can be obtained. It provides a possibility to prepare surface functionalized material.

Polypropylene (PP) is a non-polar polymer with good resistance against chemical reagent and mechanical abuse. PP has a good fiber-forming ability for textile use, but it lacks some reactive groups to be functionalized. The above-mentioned method can be a potential way to endow PP with certain surface properties. A wide variety of polymers can be paired with PP, even including liquid-crystalline, elastomer polymers, etc., and silicas have been successfully attempted to tailor their biphasic morphology. Lee et al. [13] utilized different silicas to regulate the structure of PP-liquid-crystalline polymer (LCP), and found that the filler size and surface nature affected the morphology of the LCP phase, and the compatibility between the filler and the PP matrix impacted the droplet-fibril transition significantly. Liu et al. [14] and Yang et al. [15,16] introduced different silicas into PP-elastomer blends, and detected that the addition of nanosilica has a different influence on the impact properties, resulted from the varying morphology of the blends, such as a decreased dispersed domain or SiO_2_-surrounded droplets. Elias et al. [17,18,19] used different kinds of silicas to tailor the structure of PP-PS and PP-EVA blends, and migration could be observed after processing the melt, mainly resulting from shear-induced movements and collisions with the dispersed phase. It was discovered that the different localization of the filler played different roles in stabilization, and different silicas endow the morphology of polymer blends with tremendous feasibilities.

Thermoplastic polyvinyl alcohol (PVA), as polar polymer, is immiscible with PP, and it can be removed in warm water as a sacrificial phase. Nowadays, few studies of PP-PVA biphasic system [20,21,22,23] have been done, in which glycerin is introduced into PVA matrix as a plasticizer. As a matter of fact, new thermoplastic PVA can be melt-manufactured without any plasticizer. To the best of our knowledge, so far there are hardly any reports [23] concerning the morphological regulation of PP-PVA blends by adding nanofillers. Hence, the current work emphatically discusses the utilization of silica nanoparticles in tailoring the structure of PP-PVA blends. 

As we can see, continuity of sacrificial PVA is a critical parameter in preparing the porous material. There are some studies concerning the regulation by introducing the silicas, and different localizations lead to different consequences. For the silicas localized in the bulk polymers, Steinmann et al. [24] utilized a large quantity of hydrophilic SiO_2_-spheres for PS-PMMA system (minority of PMMA in mass), and the filler was fully selective for PMMA. It was noted that it led to a promotion of PMMA continuity, and the dispersed domains were more elongated and irregular. Lee et al. [25] used hydrophobic nanosilica on the PP-polyolefin (POE) elastomer blends (mass ratio of 50/50), in which silicas were located in the PP phase, resulting in a drop of POE continuity. For the nanoparticles restricted at the interface, Filippone et al. [26] used hydrophobic SiO_2_ to modify the HDPE-PEO blend (minority of PEO in mass), and the interface-located silica would decrease the PEO continuity degree, for the sizes of PEO phase are downsized irrespective of its continuity. It can be interesting to compare the relationships between the continuity degree and different localizations of the fillers.

For the morphology regulation of the PP-PVA blends, two approaches were exploited. First, blends with three different mass ratios of PP/PVA (70/30, 60/40, 50/50 (wt %/wt %)) were investigated. Second, efforts were made to use different kinds of silicas to analyze the impacts to the microstructure evolution, in which their mass ratio was 70/30 (PP to PVA). Additionally, efforts were also made to develop blends with interface-distributed silicas, thus, it can further prove the feasibility of fabricating surface-modified PP material. In most cases, melt manufacturing of polymer blends should undergo the extrusion process. Hence, we put research emphasis on the extrudates of the polymer blends. The PVA continuity may help to speculate the localization of fillers, due to their different impacts to the biphasic morphology. Therefore, the cross- and longitudinal sections were observed by scanning microscopy analyses (SEM), and the relationship between PVA continuity and their specific topography was investigated. Additionally, rheological analysis was also carried out, which help to better understand the evolution of the structure of PP-PVA blends.

## 2. Materials and Methods

### 2.1. Materials

Polypropylene (PP) (PPH 9069) was a commercial product from Total Enterprise (Courbevoie, France). Its melting temperature is 168 °C, with melt flow index (MFI) of 15 g/10 min (200 °C, 2.16 kg). Polyvinyl alcohol (PVA) (OKS-8112P) was kindly supplied by Nichigo Gohsei (Osaka, Japan). Its melting temperature is 179 °C, and the MFI is 30 g/10 min (200 °C, 2.16 kg).

Three different silica nanoparticles were utilized in the experiments. The hydrophilic unmodified silica nanoparticles (Sigma-Aldrich^®^ S5505, St. Louis, MO, USA, denoted as S5505) with 14 nm of nanoparticle size were purchased from Sigma-Aldrich Company, and its surface area is 200 m^2^/g. Meanwhile, two commercialized fumed silicas (Aerosil^®^ R816, Aerosil^®^ R972, denoted as R816, R972) manufactured by Evonik Company (Essen, Germany) were likewise utilized. R816 and R972 were prepared from one kind of unmodified silica via silanization, surface treated with hexadecylsilane and dimethyldichlorosilane, respectively, showing different levels of hydrophobicity. Their surface areas are 190 and 110 m^2^/g, respectively, and the nanoparticle size 12 and 16 nm. The aforementioned materials were received for use without any pretreatment.

### 2.2. Sample Preparation

All of the materials were melt-mixed into blends by means of a co-rotating intermeshing twin-screw extruder (Thermo Haake, screw diameter = 16 mm, *L*/*D* = 25). Different ratios (50/50, 60/40, 70/30 (wt %/wt %)) of PP and PVA were dried in the oven at 80 °C for at least 12 h to eliminate the residual moisture. The screw rotational speed was maintained at 100 rpm. The extruder contains five heating zones, of which the temperatures were 160 °C/170 °C/180 °C/190 °C/200 °C, respectively. The material in the form of rods was obtained and rapidly cooled down by airstream, followed by cutting pelletization for further characterizations. Depending on the mass ratio differences, the pellet samples are denoted as PP_x_-PVA_y_, where the mass ratio of PP and PVA is *x* wt %/*y* wt %. For instance, PP_60_-PVA_40_ represents the pellets composed of 60 wt % of PP and 40 wt % of PVA.

In order to explore the effects of different fumed silicas, three kinds of silicas were introduced into PP-PVA blends. The same condition of extrusion in processing temperature and screw rotational speed was adopted. The mixing procedures were segregated into two steps. Firstly, the silica nanoparticles were precompounded with PP. Secondly, the prepared blends were extruded again with PVA, thus, the blended PP-PVA-silica samples were obtained. The mass fraction of silica in the final blends was 1 wt %, and meanwhile in order to better observe the regulation of the continuity of PVA by the silicas, a relatively lower fraction of PVA was selected. Therefore, the mass ratio of PP and PVA was fixed at 70 wt %/30 wt %. Similarly, the samples were cooled down and cut into pellets. The samples containing hydrophilic S5505 silicas and hydrophobic R816, R972 silicas are denoted as PP_70_-PVA_30_-S5505, PP_70_-PVA_30_-R816, and PP_70_-PVA_30_-R972.

### 2.3. Selective Phase Extraction Experiment

For the PP-PVA blends, PVA is soluble in warm water, and on the other hand, water has no implication to the PP matrix. The determination of PVA continuity with warm water in the immiscible blends gives a good approximation of the co-continuity extent [27]. Approximately 4.0 g of blends and some filter papers were dried for 12 h at 50 °C and weighed. Then, tested blends were immersed in 100 mL of distilled water under magnetic stirring for 5 h at 80 °C to extract PVA phase. The entire solution was poured onto a filter paper, and the samples were rinsed with warm water to dilute the peripheral PVA solution. The sample with filter paper was dried overnight in the oven at 50 °C to get rid of water. The PVA continuity degree was calculated as the fraction of the removed PVA in the PVA part in the blend. Therefore, the PVA continuity degree can be calculated by Equation (1). The values were recorded for at least two times and the average values were taken.

(1) $$\;\mathrm{PVA}\;\mathrm{continuity}\left(\%\right)=\frac{W_{i}-\left(W_{r+f}-W_{f}\right)}{W_{i}\times \omega_{\mathrm{PVA}}}\times 100\;$$

where *W*_i_ is the initial weight of the polymer blends before extraction, *W*_r+f_ is the total weight of the dried residual polymer and filter paper, *W*_f_ is the weight of the dried filter paper before use, ω_PVA_ is the mass fraction of PVA in the blends.

### 2.4. Microscopy Characterization and Image Analyses

The extrudates were dipped into liquid nitrogen to be frozen and fractured by a razor blade in cross- and longitudinal sections. SEM (Hitachi S4700, Hitachi, Tokyo, Japan) was utilized to investigate the cryogenic fracture surfaces. The accelerating voltage is 6.0 kV. The dispersed PVA particles sizes were determined by ImageJ software (National Institutes of Health (NIH), Bethesda, MD, USA), and more than 300 PVA particles were measured in SEM micrographs of longitudinal fracture surfaces.

**Scheme 1:**
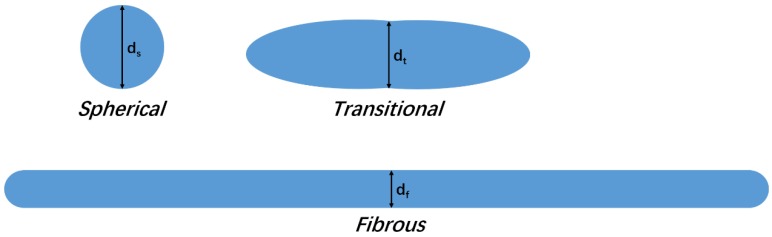
The diagram of diameter measurement of PVA nodules of different shapes.

Form factor (ff) is a measure of the deviation of a finite shape from circularity (=4 × π × area/perimeter^2^), and the parameter can be a powerful evidence in determining the PVA continuity. When ff is close to 1, it tends to be a perfect circle; when ff is approaching 0, it illustrates that the perimeter is extremely tremendous and the shape is irregular. Herein, in order to observe the evolution of the PVA shape distribution, the PVA nodules were distributed into three classes, spherical (ff > 0.60), transitional (0.60 > ff > 0.15), fibrous nodules (ff < 0.15). Among the several hundreds of nodules, the number of spherical, transitional, and fibrous nodules are *N_s_*, *N_t_* and *N_f_*, respectively. The diagram of diameter measurement of PVA nodules of different shapes is displayed in Scheme 1. For spherical nodules, the diameter was estimated as $d_{si}=\sqrt{4A_{si}/\pi }$, where *A_si_* was the area of the *i*-th spherical nodule. The average diameter of the spherical nodules was calculated as $D_{sv}=\sum_{i=1}^{N_{s}}{d_{si}}^{4}/\sum_{i=1}^{N_{s}}{d_{si}}^{3}$. For transitional nodules, the diameter of the middle part was measured as *d_ti_* for the *i*-th drop. The average diameter of the transition nodules was evaluated as $D_{\mathrm{tn}}=\sum_{i=1}^{N_{t}}d_{ti}/N_{t}$. As for the fibrous nodules, the diameter was estimated as $d_{\mathrm{fi}}=A_{\mathrm{fi}}/L_{\mathrm{fi}}$, where *A*_fi_ and *L*_fi_ were the area and axial length of the *i*-th fibrous drop. Similarly, the average diameter of the fibrous nodules was estimated as $D_{\mathrm{fn}}=\sum_{i=1}^{N_{f}}d_{fi}/N_{f}$.

Transmission electron scanning microscopy (TEM) was performed on the cross-section of the rods. The cross-section was obtained by slicing the sample with the aid of liquid nitrogen to the thickness of tens of nanometers. The microstructure of the samples was observed by Philips CM100 transmission electron microscope.

### 2.5. Rheological Measurement

The melt rheological behaviors were carried out by a TA rotational rheometer (AR 2000ex, TA Instruments, New Castle, DE, USA) in the mode of oscillatory shear flow. The diameter of the parallel plate was 25 mm and the gap was set at 1.5 mm. The frequency sweep was ranged from 0.01 to 628 rad/s. All measurements were conducted at 190 °C.

### 2.6. Surface Tension Measurement

There is a tight relationship between the surface tension and contact angle of materials. The contact angles between probe liquids and polymers were monitored by a sessile drop apparatus named “Digidrop” manufactured by GBX. In order to ensure a smooth surface for contact angle tests, hot pressing technology was utilized to make molded plates. Neat PP or PVA pellets were placed between Teflon plates with heating over their melting temperatures without pressure. Then a high pressure between the two plates (40 bars) was offered and lasted for some time. Afterwards, the pressure was maintained and the plates were water-cooled to the room temperature. After the stress reliever of the plates, the neat polymer plates with smooth surfaces were generated for contact angle measurement. 

The contact angles of the liquid on the polymer surface enable us to obtain the polar and dispersive contributions to the surface tension of PP and PVA by the method of Owens and Wendt [28] (Equation (2)):

(2) $$\sqrt{\gamma_{s}^{d}\gamma_{l}^{d}}+\sqrt{\gamma_{s}^{p}\gamma_{l}^{p}}=0.5\gamma_{l}\left(1+cos\theta \right)\;$$

where *θ* is the contact angle, and $\gamma_{l}$ is the liquid surface tension (mN/m), $\gamma_{s}^{d}$ and $\gamma_{s}^{p}$ are the dispersed and polar component of the solid surface (mN/m), $\gamma_{l}^{d}$ and $\gamma_{l}^{p}$ are the dispersed and polar component of the liquid surface tension (mN/m). Two probe liquids were utilized, one apolar liquid (diiodomethane, *γ* = 50.8 mN/m, *γ^p^* = 2.3 mN/m, *γ^d^* = 48.5 mN/m) and one polar liquid (distilled water, *γ* = 72.8 mN/m, *γ^p^* = 51.0 mN/m, *γ^d^* = 21.8 mN/m) [29].

## 3. Results and Discussions

### 3.1. Influence of the Mass Ratio of PP-PVA Blends

The dual-phase continuity can be strongly affected by the ratio of the two polymers [30]. In this study, PVA is selected as the minority phase, and the fraction of PVA ranges from 30 to 50 wt %. An appropriate ratio of PP to PVA is beneficial for constructing a favorable scaffold microstructure.

#### 3.1.1. Morphology of the Polymer Blends

**Figure 1:**
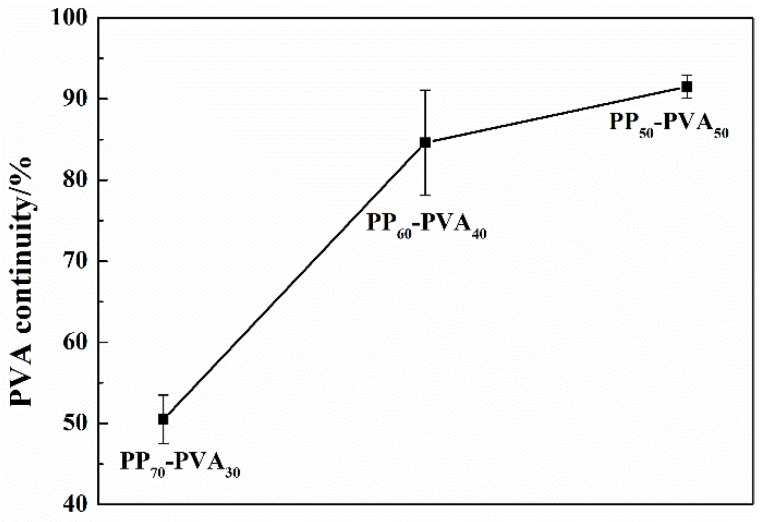
PVA continuity degree of the PP-PVA blends with different mass ratios of polymers.

The selective extraction experiment provides a facile method to evaluate the co-continuity extent of the polymer blends. Figure 1 graphically indicates the PVA continuity degree of the PP-PVA blends with different mass ratios. For PP_70_-PVA_30_, the PVA continuity is 50.5% ± 3.0%, which indicates that only half of PVA can be removed. With the content of the PVA increasing, the PVA continuity degree increases violently, resulted from the change of the microstructure of PP-PVA.

**Figure 2:**
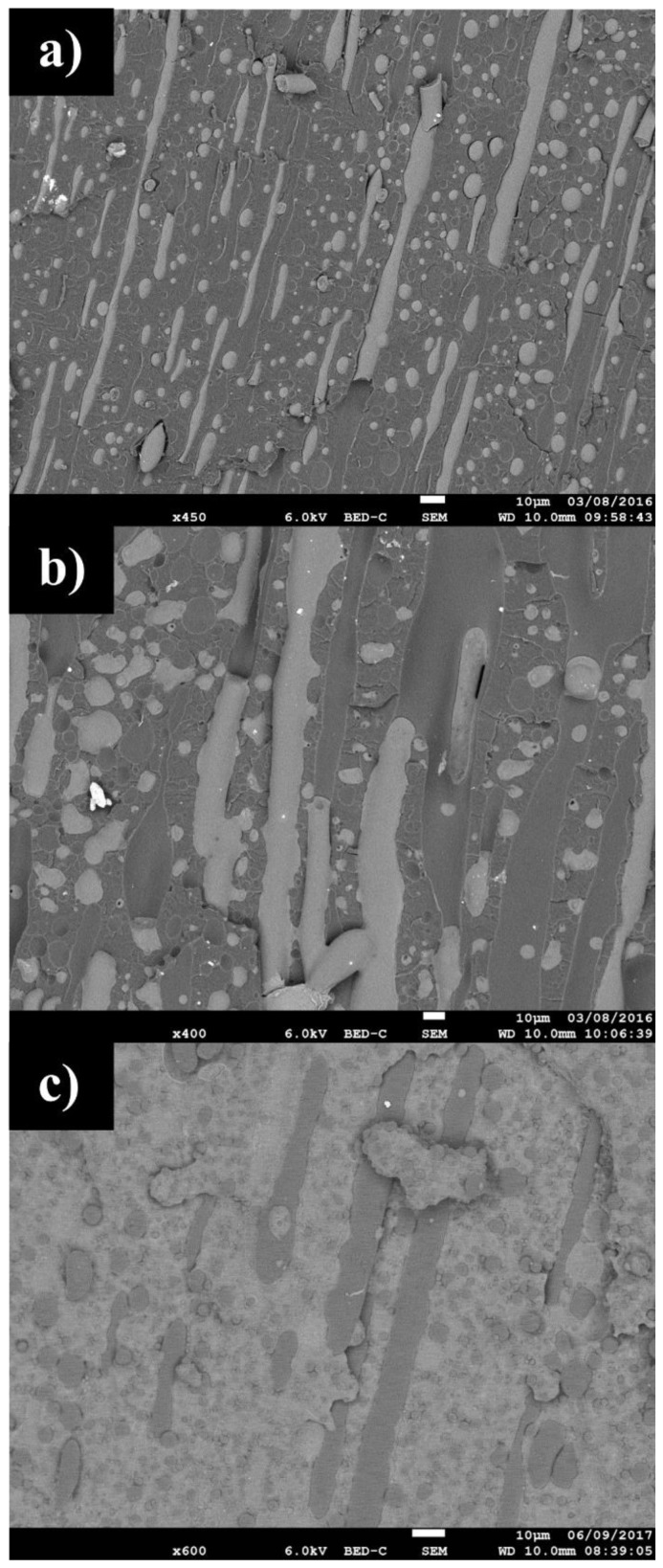
SEM images of the pellets with different mass ratios in longitudinal direction: (**a**) PP_70_-PVA_30_; (**b**) PP_60_-PVA_40_; and (**c**) PP_50_-PVA_50_.

In order to intuitively observe the evolution of the biphasic microstructure, SEM observation was also given. Cross-sections of the extrudates perpendicular to extrusion direction have been observed, all the projections of PVA nodules presents circular. Thus, the longitudinal section of the extrudates can render the information of their biphasic morphology quite well. Figure 2 shows the SEM images of the longitudinal section of the pellets with different mass ratios of PP and PVA. In order to distinguish PP and PVA, a backscattered electron detector (BSE) was applied. The light-colored part in the SEM images is PVA and the dark-colored part is PP. Figure 2a is the longitudinal direction observation of PP_70_-PVA_30_ pellets. PVA forms isolated ellipsoidal and spherical droplets inside the PP matrix. Apart from shear stress, some extensional stress also exists during the extrusion process, which leads to the elongation of the PVA nodules. As the content of PVA increases, some PVA forms an interconnected structure instead of isolated droplets, which gives the evidence that its morphology is approaching co-continuous structure. Its high PVA continuity (84.6% ± 6.5%) also gives the same implication. Furthermore, their sizes and shapes are quite diverse. With the content of PVA further increasing, the PVA continuity is even higher (91.5% ± 1.4%), and it can be assumed that nearly all of PVA becomes approachable. Based on the SEM image of PP_50_-PVA_50_, it illustrates that the roles of PP and PVA phases are reversed. PVA phase becomes highly continuous and PP phase is fenced off by PVA phase. This gives an inference that the structure was evolved from the PP matrix-PVA dispersed structure to the PVA matrix-PP dispersed structure, which experienced a phase inversion.

By altering the PVA content, the microstructure of PP-PVA blends experiences a significant change. The SEM observation fits well with the PVA continuity results. Thus, the ratio tailoring upon PP-PVA blends dominantly governs the biphasic microstructure.

#### 3.1.2. Rheological Characterization

**Figure 3:**
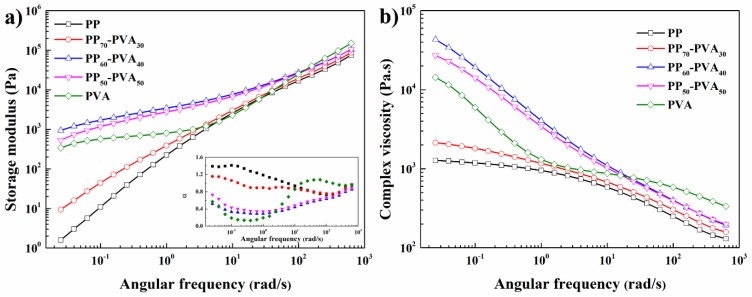
Rheological behaviors of neat PP, PP_70_-PVA_30_, PP_60_-PVA_40_, PP_50_-PVA_50_, and neat PVA. (**a**) Storage modulus and the slope of the storage modulus α(ω); and (**b**) complex viscosity.

Rheological behaviors of polymer blends are tightly relevant to the microstructure evolution of biphasic polymers [31,32]. Steinmann et al. [26] considered that the rheological evaluation is sometimes even more suitable for the determination of phase inversion concentration than morphological evaluation, for it reflects the three-dimensional bulk properties rather than the two-dimensional images. It also provides a plain way to discover the characteristic of polymers, from the perspective of the shape relaxation of the dispersed phase. The viscoelastic behaviors of neat polymers and their blends were measured, and the storage modulus and complex viscosity are illustrated in Figure 3. Aimed at quantitatively obtaining the trends of these curves, the local slope of the storage modulus, α(ω) (= ${\frac{\partial \mathrm{logG}\prime }{\partial \log\omega }|}_{\omega }$) was also calculated and shown in the lower right corner of Figure 3a. α values of any materials are between 0 and 2, and the lower limit 0 represents the response of a purely elastic solid, and the upper limit 2 stands for the terminal behavior of a Maxwellian fluid [33]. At the low frequency (0.025 rad/s) the value of α_PVA_ and α_PP_ are about 0.6 and 1.4, respectively. PP behaves like a Maxwellian fluid, while PVA demonstrates more distinct features of an elastic solid. It is worth noting that there is a Newtonian plateau of complex viscosity in PP, while there is no such phenomenon in PVA and, instead, there is a sharp slope. The solid-like behavior may have resulted from the existence of dynamic hydrogen bonds between PVA molecules, which played the role of dynamic physical crosslinking points; thus, the relaxation of PVA molecules is limited [34].

For PP_60_-PVA_40_ and PP_50_-PVA_50_, the storage moduli are even higher than that of sole PVA and PP, which gives evidence that the cause not only led from the bulk polymers. The extra part of the storage modulus is also owing to the shape relaxation of the dispersed phase. In detail, the secondary plateau is dominantly led by the size and the amount of dispersed phase, and its width is related to the size distribution of the drops [35]. When the fraction of PVA increases from 40 to 50 wt %, the storage modulus decreases slightly, even though PVA has a more excellent solid-like properties. It is due to the phase reversion of PP and PVA, proving that the existence of co-continuous structure lies in the blends, in which the PVA content is near 40–50 wt %. The evolution has been observed by SEM measurement. In addition, as for the complex viscosity, there are no Newtonian plateaus in PP_60_-PVA_40_ and PP_50_-PVA_50_, mainly resulting from the enormous amount of continuous PVA.

In terms of the α values, the rheology behavior of PP_70_-PVA_30_ verges upon that of PP, and the α_PP70-PVA30_ is reduced slightly to 1.2. For PP_60_-PVA_40_ and PP_50_-PVA_50_ blends, the α values are lower with the values of 0.5 and 0.7. The slope of storage modulus (α) is sensitive to the degree of interconnectivity in materials with network structure [36]. The solid-like behavior of PP60-PVA40 is even more evident than PVA, although the fraction of PVA is only 40 wt %. The further enhanced elasticity is due to the biphasic structure approaching co-continuous structures [35].

### 3.2. Tailoring the Microstructure via Introducing Silicas with Different Wettability

The incorporation of nanoparticles can endow the bulk polymers with additional properties. They also play an important role in tailoring the microstructure of the biphasic materials. Apart from tailoring the ratio of PP and PVA, this part will also take silica particles, for example, to discover the microstructure diversification of PP-PVA blends. PP_70_-PVA_30_, the classic matrix-dispersed structure is selected to be investigated. The mass ratio of PP to PVA is kept at 70 to 30 wt %, and 1 wt% (overall) of fumed silicas are introduced into the blends. Three typical silicas with different wettability are hydrophilic S5505, partially hydrophobic R816, and highly hydrophobic R972.

#### 3.2.1. Prediction and Confirmation of Silica Localization 

Different silicas will localize in different domains of polymers. The localization is controlled by several factors, including kinetic and thermodynamic influences. When the processing time is long and the shear force is strong enough for equilibrium, the localization of particles is governed by thermodynamics [17]. The localization of the fumed silicas can be predicted by the Young’s model (Equation (3)) [37]:

(3) $$\omega_{A-B}=\frac{\gamma_{Si-B}-\gamma_{Si-A}}{\gamma_{A-B}}\;$$

where *γ_si_*_-*i*_ represents the interfacial tension between the fumed silicas and the polymer *i*, and *γ_A-B_* represents the interfacial tension between the two biphasic polymers.

As for the localization of silicas, when *ω_A-B_* < −1, silicas will be localized in phase B; when *ω_A-B_* > 1, silicas will be localized in phase A; and if *ω_A-B_* is of other values, silicas will be localized at the interface between the two polymers.

The surface tensions of polymers and fumed silicas at 25 °C and 200 °C are illustrated in Table 1. The related results were normally conducted at room temperature (25 °C). However, the surface tension is temperature dependent, and the pellets were extruded at a high temperature (160–200 °C). Therefore, it would be better to carry out the values at the extrusion temperature. For the tested polymers, the relationship of Guggenheim [38] was applied to obtain the *γ* at 200 °C (Equation (4)):

(4) $$\gamma =\gamma_{0}(1-\frac{T}{T_{c}}{)}^{11/9}\;$$

where $\gamma_{0}$ is the surface tension at 0 K; *T*_c_ is the critical temperature, which can be consulted in the literature [39]; *T* is the temperature of the polymers. For the fumed silicas, the *γ* value is cited from the related representative literatures. It is worth noting that the surface energy of S5505 is derived by the related contact angles of one kind of typical bare fumed silica. Elias et al. [18] proposed that for silica particles, the surface tension can be estimated by using the constant rate *d*γ/*d*T as −0.1 mN m^−1^ K^−1^. 

Yan et al. [40] found that although R816 has been modified with hexadecylsilane, it can also be easily dispersed into water, still behaving relatively hydrophilic, which is reflected in its surface energy. It shows increasing hydrophobicity followed by S5505, R816, and R972 silicas.

For the systems at 200 °C, estimation of the interfacial tension can be achieved via Equations (5) and (6). Equation (5) is the geometric average based on Fowkes theory, which is more suitable for filled polymer systems with high differences in surface energy. Equation (6) is the harmonic average based on Wu theory, which is more accurate for neat polymer blends with small differences in surface energy [41,42]. Both geometric and harmonic averages of interfacial energy (Equations (5) and (6)) between polymers and silicas are listed in the Table 2:

(5) $$\gamma_{1-2}=\gamma_{1}+\gamma_{2}-2\sqrt{\gamma_{1}^{d}\gamma_{2}^{d}}-2\sqrt{\gamma_{1}^{p}\gamma_{2}^{p}}\;$$

(6) $$\gamma_{1-2}=\gamma_{1}+\gamma_{2}-\frac{4\gamma_{1}^{d}\gamma_{2}^{d}}{\gamma_{1}^{d}+\gamma_{2}^{d}}-\frac{4\gamma_{1}^{p}\gamma_{2}^{p}}{\gamma_{1}^{p}+\gamma_{2}^{p}}\;$$

The wetting parameters ω_PP-PVA_ can be obtained by Equation 3. Regarding PP as phase A and PVA as phase B, both of the geometric and harmonic averages of ω_PP-PVA_ were calculated and are shown in Table 3. ω is calculated less than −1 for both PP-PVA-S5505 and PP-PVA-R816 samples, which predicts that at thermodynamic equilibrium, the fumed silicas will be preferentially located inside the PVA phase. As for PP-PVA-R972, the value of ω_PP-PVA_ is 0.72 in geometric mean and 0.90 in harmonic mean, of which both indicate that R972 silica tends to be limited at the interface of PP, while for extrusion, complete thermodynamic equilibrium may be difficult to achieve, because the processing time is short [17]. However, due to the enormous shear force of the twin screws in the extrusion machine, the thermodynamical prediction still offers a strong preference to predict the tendency of the nanofiller migration.

**Figure 4:**
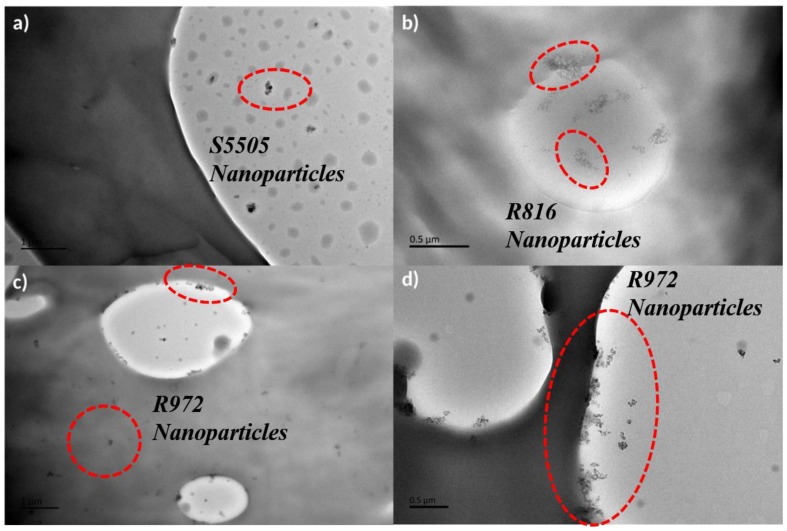
The TEM observation of the PP-PVA blends with silicas. (**a**) PP_70_-PVA_30_-S5505; (**b**) PP_70_-PVA_30_-R816; (**c**) PP_70_-PVA_30_-R972; and (**d**) PP_70_-PVA_30_-R972 (higher magnification).

In order to confirm the localizations of different silicas, the TEM observation of the cross-section of the extrudates was carried out. The definite localizations of nanoparticles are summarized in Table 4. Representative TEM images of three blends are enclosed in Figure 4. Due to the lower fraction of PVA, spherical PVA domains can be distinguished in the PP matrix. Hydrophilic unmodified S5505 silicas are trapped in the PVA domains, because of the strong affinity between S5505 and PVA. The definite distribution of S5505 silicas is identical with the prediction. R816 nanoparticles are predicted to be localized within the PVA nodules, however, the silicas are found not only in the PVA phase, but also at the interface of PP and PVA. The blending procedure is decided as pre-blending silica particles in PP matrix and blending PVA and the silica-filling PP pellets. Therefore, owing to the kinetics of the silica particles, R816 transferred from less preferred PP phase to the PVA phase, passing through the interface, and a tiny number of silicas appear at the interface. 

The hydrophobic R972 silica is localized exclusively at the biphasic interface, while a small amount of R972 silicas can also be found within the bulk phases. Given the wetting parameter ω_PP-PVA_ (near 1) of R972 and the kinetic factors, it is not difficult to explain why the silicas are also distributed in the PP phase. As for the silicas with PVA phase, it may result from the modification inhomogeneity of R972 silicas. Nevertheless, it is inspiring that a large quantity of silicas is distributed at the interface and form a partial thin layer, although only 1 wt % of silicas are involved in the blends. It can be expected that if a higher quantity of R972 silicas are incorporated, the interface enrichment behavior of silicas would be enhanced. If PVA is removed afterwards, surface-modified PP can be obtained.

#### 3.2.2. Morphology of the Polymer Blends

**Figure 5:**
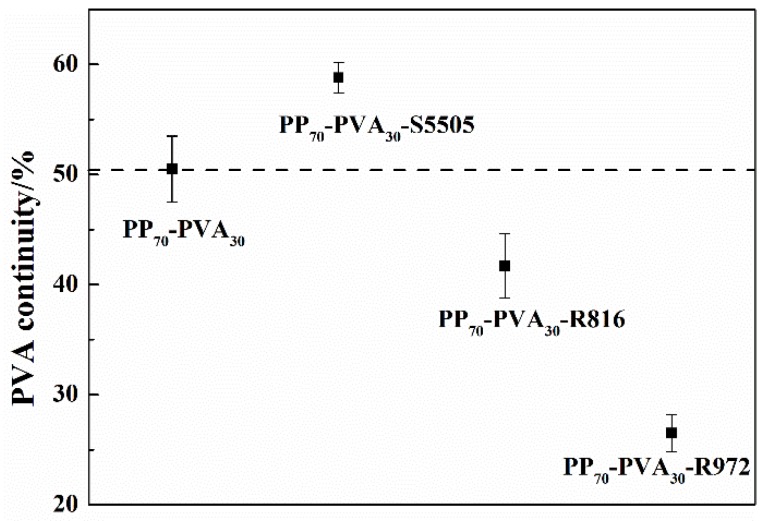
PVA continuity degree of the PP-PVA blends with 1 wt % of silicas with different hydrophobicity.

As we know, the heterogeneously distributed nanoparticles will affect the morphology evolution. Thus, the biphasic morphology investigation of the three blends is concentrated on. First of all, the polymer blends were selectively extracted by water to test the PVA continuity degree, and the results are displayed in Figure 5. The PVA continuity degree of PP_70_-PVA_30_ is 50.5% ± 3.0%, and it maintains below 60% with the addition of silicas, implying that PP and PVA still have a matrix-dispersed structure. However, the PVA continuity ranges a great deal, which indicates that the addition of silicas will affect the interconnection of PVA significantly. After introduction of S5505 nanoparticles, the PVA continuity degree increases slightly to 58.8% ± 1.4%. If the modified silicas R816 and R972 are incorporated into the blends, the PVA continuity will decrease to 41.7% ± 2.9% and 26.5% ± 1.7%, respectively. It is chiefly ascribed to the different localization of the fumed silicas.

**Figure 6:**
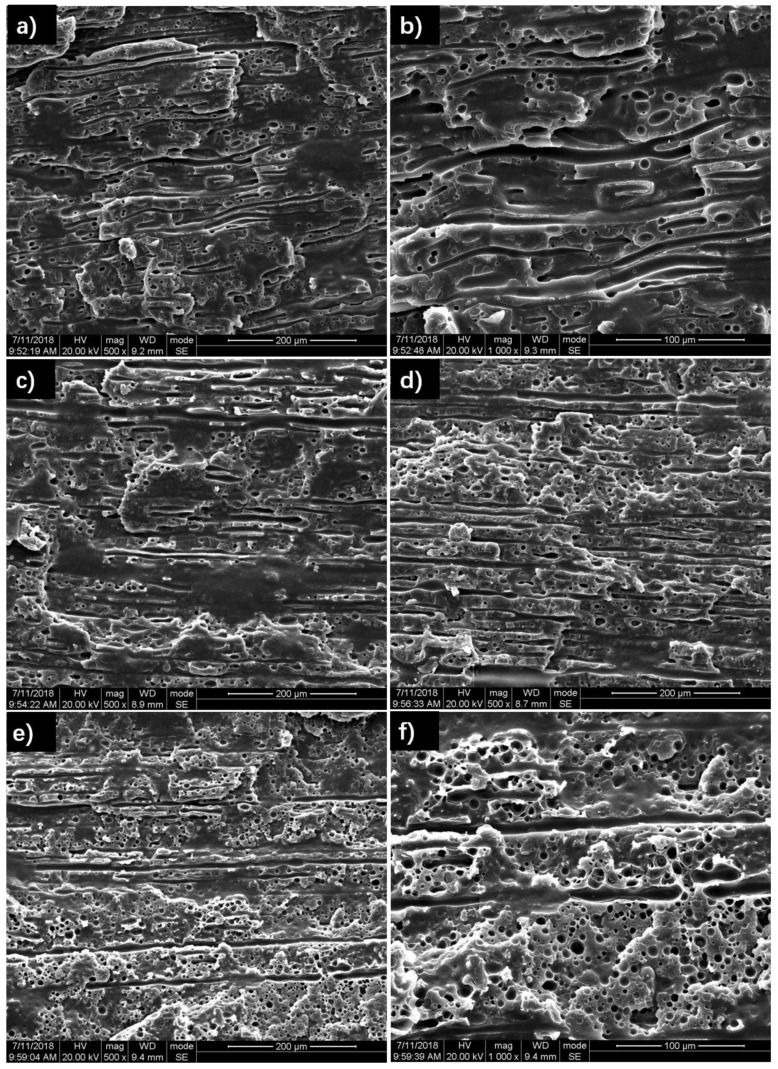
SEM images of the longitudinal section of the extrudates. (**a**,**b**) PP70-PVA30; (**c**) PP70-PVA30-S5505; (**d**) PP70-PVA30-R816; and (**e**,**f**) PP70-PVA30-R972.

To systematically investigate the morphology evolution of the blend extrudates, the SEM observation was carried out. The shapes of the PVA nodules from the cross-section are identical as circles, of which the SEM results are not presented in consequence. The SEM images of the longitudinal section of PP_70_-PVA_30_ extrudates with and without fillers are shown in Figure 6. The PVA nodules are classified into 3 sorts, spherical, transitional, and fibrous, and the average diameters of the three kinds of PVA nodules were counted. The methodology of measurement has been stated in Section 2.4. The fiercely deformed nodules (transitional and fibrous) determine the PVA continuity of the blends, which make the PVA interconnected. In addition, the volume of one nodule can be viewed as the integral of the tremendous projections of every layer, thus, the projection fraction of the classified PVA nodules was totaled, and it can represent the proportion of the three components to some extent. The average diameter and projection fractions are illustrated in Table 5. 

For PP_70_-PVA_30_ blend, transitional PVA is the major component in the PVA nodules (41.9%). The dominant fraction goes to the fibrous structure, and the diameter of fibrous PVA is elevated after the addition of nanofillers. It suggests that the addition of fillers will help the formation of fibrous PVA. For the spherical and transitional PVA, the localization of the silicas makes the diameter and fraction quite varied. For S5505 silicas, it centralizes within the PVA phase. As a result, the diameter of spherical and transitional PVA increases. More spherical and transitional PVA will transform into fibrous PVA. The elongation and irregularity will contribute to the increment of the PVA continuity. It may be caused by the slowdown of destructive processes like fibril breakup by filling [24].

For hydrophobic R972 silicas, the dominant position of silicas goes to the interface. The enlarged SEM images of PP_70_-PVA_30_ and PP_70_-PVA_30_-R972 are enclosed in Figure 6b,f, respectively. It can be seen clearly that there are more spherical nodules instead of transformed ones. From Table 5, the fraction of transitional PVA is reduced from 41.9% to 15.0% and instead the fraction of spherical PVA is increased from 22.1% to 40.0%. What’s more, the R972 silicas lead to a significant reduction of diameter of spherical and transitional PVA. It can be ascribed that these interface-located silicas can act as steric hindrance or exert surface energy effects, preventing the coalescence of PVA nodules [17].

As for R816 silicas, the TEM observation has illustrated that the silicas are located within the PVA phase and at the interface. Although the silicas inside the PVA phase can contribute to the increment of PVA continuity, the prevention effect of interface-located silicas in PVA coalescence cannot be ignored. As a result, the diameter of spherical and transitional PVA increases, while more transitional PVA is transformed into spherical PVA. As a result, it gives rise to the slight reduction of PVA continuity. Silicas with different localizations endow PVA continuity with diversities by tuning the biphasic morphology. Conversely, PVA continuity also gives clues about the silica localizations.

#### 3.2.3. Rheological Analyses

**Figure 7:**
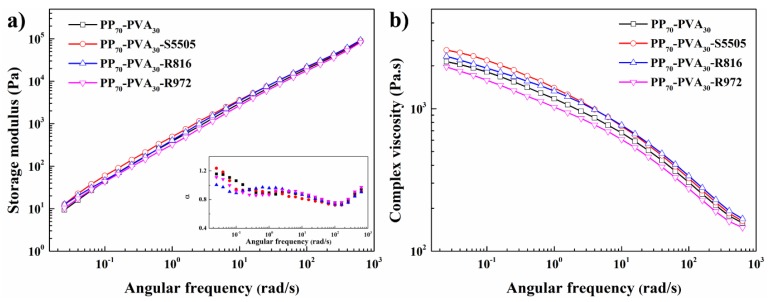
Rheological behaviours of PP_70_-PVA_30_, PP_70_-PVA_30_-S5505, PP_70_-PVA_30_-R816 and PP_70_-PVA_30_-R972. (**a**) Storage modulus and α(ω); and (**b**) complex viscosity.

The linear viscoelastic behavior of the blends was examined by the rheological analyses. The results are illustrated in Figure 7. Storage modulus and α(ω) curves are respectively displayed in Figure 7a, and the complex viscosities of the polymers are given in Figure 7b. For the additional fraction of silica is only 1 wt %, *G*’ curves of the blends with silicas are not changed obviously. However, the *G*’ curves present subtle differences, which can still offer some evidence about the evolution of the microstructure. Among the blends, their high frequency response is almost the same, which is only controlled by the relaxation process of the polymer chains, while for the low-frequency area the storage modulus shows some differences. Upon the addition of silica nanoparticles, all of the α(ω) values at the low frequency are almost decreased. The extra elasticity should be confirmed by many factors, and different localizations of nanoparticles make a different contribution. For PP_70_-PVA_30_-S5505, it may be resulted from the increasing interconnectivity of the PVA nodules; while for PP_70_-PVA_30_-R816 and PP_70_-PVA_30_-R972, although the shapes and sizes are refined leading to a decrease of elasticity, the solid character of the interface filled with silicas gives a stronger contribution. It leads to an even lower α(ω) value at the low frequency area compared with PP_70_-PVA_30_ blend. The addition of silicas has a certain effect upon the complex viscosities of the blends. After the addition of S5505 or R816, the complex viscosity will be enhanced; after the introduction of R972, its complex viscosity will be weakened. The alteration may be influenced by the biphasic morphology, which is determined by the localization of silicas.

## 4. Conclusions

In this work, the microstructure of PP-PVA was tailored by two methods. One method is to alter the ratios of two polymers; the other method is to introduce 1 wt % of silica nanoparticles. The PVA continuity investigation combined with microscopy observation was conducted to track the microstructure evolution, correlated by the rheological tests. It indicates that the biphasic microstructure experiences a significant change by altering the PVA content. The structure was evolved from PP matrix-PVA dispersed structure (PP_60_-PVA_40_) towards PVA matrix-PP dispersed structure (PP_50_-PVA_50_). For rheological analysis, the variation in storage modulus of blends also provides an evidence for the phase inversion.

The addition of silicas likewise brings a very large impact on the biphasic morphology of PP-PVA. Hydrophilic S5505 and partially hydrophobic R816 were predicted to be localized in PVA phase. The prediction about S5505 was confirmed, however, R816 was also found to distribute at the interface. R972 was forecasted to be concentrated at the interface. The dominant localization at the interface was verified, although there was still a small amount existing within the bulk phases. Generally speaking, the definite localizations are basically consistent with thermodynamical prediction.

The different localization gave rise to the distinctive biphasic morphology of PP-PVA. The PVA continuity degree of PP_70_-PVA_30_ is increased after the introduction of S5505, and it is decreased instead because of the introduction of R816 or R972. S5505 conduces to the increment of PVA size and its fraction in fibrous form, which is beneficial for the elevation of PVA continuity. It may be caused by the slowdown of destructive processes from the S5505 silicas within the PVA phase. R972 is in favor of forming spherical PVA nodules in the polymer blends and refining the sizes of PVA, which leads to the reduction of PVA continuity. It can be ascribed that these interface-located silicas can act as a steric hindrance or exert surface energy effects, preventing the coalescence of PVA nodules. R816 is localized within PVA and at the interface, and synthetically impacts the biphasic morphology. The rheological analysis revealed that upon the addition of silica nanoparticles, all the polymer blends have an extra elasticity due to different reasons.

R972 can be an example of a surface modification material from PP-PVA blends. Beyond that, the different localization will give different impact to the morphology, mainly changing the distribution and size of PVA with different shapes. As a result, it will be inevitably reflected in the PVA continuity. Conversely, it provides a referential route to predict the localization of the silica fillers by the change of PVA continuity. For instance, if the silicas are required to localize at the interface, the PVA continuity is expected to be decreased, which gives a preliminary judgment to rapidly screen out suitable nanoparticles.

## Figures and Tables

**Table 1:** The surface tension of polymers and fumed silicas.

Component	Surface Tension (mN/m)						*γ_0_*	Tc (K) ^a^	−*d*γ/*dT* (mN m^−1^ K^−1^)
	25 °C			200 °C					
	*γ*	*γ^d^*	*γ^p^*	*γ*	*γ^d^*	*γ^p^*			
PP	24.7	24.5	0.2	16.4	16.3	0.1	40.0	914	-
PVA	60.2	32.6	27.6	41.0	22.2	18.8	95.5	948	-
S5505 ^b^	91.1	0.7	90.4	73.6	0.6	73.0	-	-	0.1
R816 ^b^	88.5	0.4	88.1	71.0	0.3	70.7	-	-	0.1
R972 ^c^	32	30	2	14.5	13.6	0.9	-	-	0.1

^a^ Related critical temperatures are cited from [39]; ^b^ Values calculated from the contact angles of water and toluene with the silicas [40]; ^c^ Values cited from [37].

**Table 2:** Interfacial energy between different components (silica with polymer, polymer with polymer) using geometric and harmonic mean equations.

Component	Interfacial Energy with PP (mJ/m^2^)		Interfacial Energy with PVA (mJ/m^2^)	
	Geometric Mean	Harmonic Mean	Geometric Mean	Harmonic Mean
S5505	78.3	87.3	33.2	52.5
R816	77.7	85.8	33.9	51.4
R972	0.5	0.9	12.5	18.3
PP	-	-	16.6	19.4

**Table 3:** Wetting parameters ω_PP-PVA_ calculated with geometric and harmonic data and the localization predictions of silica nanoparticles.

Silica Nanoparticle	Wetting Parameter (Geometric Mean)	Wetting Parameter (Harmonic Mean)	Prediction
S5505	−2.72	−1.79	PVA phase
R816	−2.64	−1.77	PVA phase
R972	0.72	0.90	interface

**Table 4:** The confirmation of the localization of nanoparticles in PP-PVA blends from TEM images.

Silica Nanoparticle	Confirmation
S5505	PVA phase
R816	PVA phase, interface
R972	PP phase, interface, PVA phase

**Table 5:** The characteristics of the microstructure of extrudates from PP-PVA blends.

Sample	Spherical PVA		Transitional PVA		Fibrous PVA	
	*D_sv_* (μm)	Projection Fraction (%)	*D_tn_* (μm)	Projection Fraction (%)	*D_fn_* (μm)	Projection Fraction (%)
PP_70_-PVA_30_	10.0	22.1	6.4	41.9	5.3	36.0
PP_70_-PVA_30_-S5505	11.8	20.6	8.5	36.0	9.2	43.4
PP_70_-PVA_30_-R816	11.4	29.3	7.1	33.0	8.1	37.7
PP_70_-PVA_30_-R972	8.3	40.0	4.7	15.0	6.4	45.0
